# Laterality index in functional MRI: methodological issues^☆^

**DOI:** 10.1016/j.mri.2007.10.010

**Published:** 2008-06

**Authors:** Mohamed L. Seghier

**Affiliations:** Wellcome Trust Centre for Neuroimaging, Institute of Neurology, UCL London, UK

**Keywords:** Functional MRI, Laterality index, Hemispheric dominance, Language system, Statistical threshold

## Abstract

In functional magnetic resonance imaging (fMRI), hemispheric dominance is generally indicated by a measure called the laterality index (LI). The assessment of a meaningful LI measure depends on several methodological factors that should be taken into account when interpreting LI values or comparing between subjects. Principally, these include the nature of the quantification of left and right hemispheres contributions, localisation of volumes of interest within each hemisphere, dependency on statistical threshold, thresholding LI values, choice of activation and baseline conditions and reproducibility of LI values. This review discusses such methodological factors and the different approaches that have been suggested to deal with them. Although these factors are common to a range of fMRI domains, they are discussed here in the context of fMRI of the language system.

## 1 Introduction

**Fig. 1:**
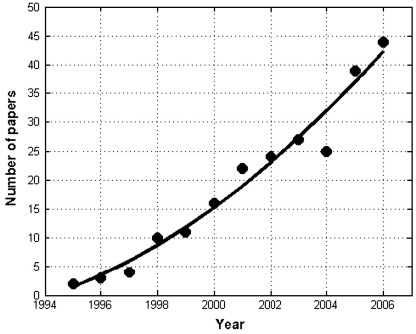
Number of papers published per year with title or abstract containing “fMRI” AND (“laterality” OR “dominance”).

Asymmetric processing of sensory, affective and cognitive information has long been one of the intriguing properties of human brain function [1–5]. Although the two hemispheres are in continual communication with each other, differences between the left (LH) and right (RH) hemispheres have commonly been reported in numerous studies with functional neuroimaging. The emergence of noninvasive functional techniques, such as functional magnetic resonance imaging (fMRI), is now providing a very interesting characterisation of this neural property, both for theoretical or clinical purposes in several domains, including language (e.g., Ref. [6]), vision (e.g., Ref. [7]), audition [8] and memory [9]. Fig. 1 demonstrates that the number of studies investigating hemispheric laterality with fMRI has increased at least linearly over the last few years, as measured by a PubMed search with “fMRI” and “laterality|dominance” as words in the title or abstract of the paper.

The most widely studied domain is undoubtedly language. Since the pioneering observations of Paul Broca, left hemisphere dominance is factually assumed for language processing. More recent studies have explored language dominance in populations with different demographic characteristics, including handedness [10,11], age [12–14], gender [15,16], multilinguism [6,17,18] and the presence of diseases [19–22]. Critically, measures of language hemisphere dominance, assessed with fMRI, have been shown to be concordant with those from other techniques, including the clinical Wada test [21,23,24], functional transcranial Doppler ultrasonography [25,26] and neuropsychological tests [27,28]. These findings have supported the usefulness of fMRI for the assessment of language dominance for clinical purposes (e.g., Ref. [29]).

The hemispheric dominance in fMRI is generally indicated by a measure called the laterality index (LI). Other groups have used the term *Asymmetry Index* (e.g., Refs. [12,14,22]); here, LI is used throughout this review. The major rational for using the LI value is to facilitate the description of hemispheric dominance from functional activation patterns because it is easier to manipulate one value per subject/contrast than thousands of voxels. However, LI assessment depends on several methodological factors that should be taken into account when interpreting LI values or comparing between subjects. Here, I attempt to present a succinct review of such factors and the different approaches that have been suggested to deal with them. These factors are valid for all fMRI domains but will be discussed here in the context of the language domain.

## 2 The LI formula

Generally, the LI value is computed using the following classic formula [23,24,30]:

(1) $$LI=f\cdot \frac{Q_{LH}-Q_{RH}}{Q_{LH}+Q_{RH}}$$

where *Q*_LH_ and *Q*_RH_ are representative quantities measured by fMRI for the LH and RH contributions, respectively. The factor *f* is a scaling factor that defines the range of LI values (i.e., LI varies continuously from −*f* for pure RH dominance to +*f* for pure LH dominance). Usually, *f* is held to 1 (i.e., LI varies between −1 to +1) or 100 (e.g., [31–33]) in which case LI varies from −100 to 100 as a percent ratio measure. Other values like 200 [27,34] or −1 [35] have also been used. Note that this formula was initially defined by assuming that all measures are positive (*Q*_LH_≥0; *Q*_RH_≥0; *Q*_LH_+*Q*_RH_>0).

Furthermore, it is interesting to examine the linearity and the sensitivity of this formula for representing differences between LH and RH contributions. Theoretically, LI can be related to the ratio between left and right contributions (*Q*_LH_ and *Q*_RH_, respectively). Specifically, the relative difference *R* between *Q*_LH_ and *Q*_RH_ can be defined as:

(2) $$R=\frac{Q_{LH}-Q_{RH}}{Q_{RH}}$$

with *R*∈[−1, +∞[. Accordingly, we can easily express LI as:

(3) $$LI=f\cdot \frac{R}{R+2}.$$

**Fig. 2:**
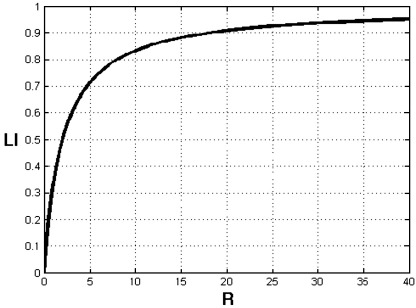
LI as a function of the relative difference (*R*) between *Q*_LH_ and *Q*_RH_ quantities (with *f*=1).

Fig. 2 shows that LI increases approximately linearly with *R* when *R* is less than 5 (i.e., *Q*_LH_ less than six times *Q*_RH_) but saturates towards a plateau for high *R* values (e.g., *R*>20). This has an important implication for the sensitivity of the LI measure to differences between LH and RH contributions. For instance, if a subject with two tests (e.g., pre- and postsurgical fMRI evaluation) shows double the increase in LH hemisphere involvement for the first than second test, then this difference will be revealed by the LI value with high sensitivity when *Q*_LH_ increases from two to four times *Q*_RH_ but very low sensitivity when Q_LH_ increases from 10 to 20 *Q*_RH_.

## 3 The nature of *Q*_LH_ and *Q*_RH_

Commonly, LI is assessed by counting the number of voxels that survive a fixed threshold within LH and RH regions of interest (e.g., Refs. [23,30]). Consequently, *Q*_LH_ and *Q*_RH_ are positive quantities. However, some studies have shown that this measure does not adequately reflect the differences between both hemispheres, as intensity differences are not taken into account. For instance, if *Q*_LH_ and *Q*_RH_ are identical, LI will be equal to zero even when voxels in the left hemisphere are statistically higher than those of the right hemisphere. To take these statistical differences into account, other authors have presented alternative measures (for more details, see Refs. [33,36]). Benson et al. [34] used signal change amplitude as a measure for *Q*_LH_ and *Q*_RH_ quantities. Practically, a histogram between the number of voxels *N* and statistics [−log(p)] is first assessed; then *Q*_LH_ and *Q*_RH_ are set equal to the weighted sum $\sum_{i}(-\log(p_{i}))\cdot N_{i}$, where −log(*p)* and *N* are the mean of the statistics (i.e., probability) and the number of voxels within the *i*^th^ bin of the histogram, respectively [34]. Therefore, LI can also reflect statistical differences between both hemispheres.

In addition, Fernandez et al. [37] have defined *Q*_LH_ and *Q*_RH_ as a sum of all *t* values above a predefined individual threshold. Practically, they defined the mean of the 5% most activated voxels within each hemisphere or region of interest (ROI) and then summed the *t* values of all voxels above 50% of this mean [37,38]. LI was then directly influenced by the *t* values in each hemisphere. Others have suggested that *Q*_LH_ and *Q*_RH_ can be quantified using the average of correlation coefficients [27], weighted *t* values [33,39,40], mean signal change [41,42], or statistical *F* values [43]. Alternatively, Baciu et al. [44] have proposed that *Q*_LH_ and *Q*_RH_ can be statistically compared by directly contrasting between correct “right side images” with “mirror images” (flipped hemispheres) of the same subjects.

One of the problems with using these statistical measures is that *Q*_LH_ and *Q*_RH_ might be negative (e.g., sum of negative *t* values), leading to some misinterpretation of LI values (e.g., Ref. [36]). In this case, users might modify their statistical threshold or move their regions of interest. It is also possible to employ a modified expression of Eq. (1) to take into account the sign of *Q*_LH_ and *Q*_RH_ quantities, as in the following formula:

(4) $$LI=f\cdot \frac{Q_{LH}-Q_{RH}}{\left|Q_{LH}|+|Q_{RH}\right|}\text{.}$$

Note also that some of these alternative measures for *Q*_LH_ and *Q*_RH_ quantities are not always superior than the standard way (i.e., *Q*_LH_ and *Q*_RH_ set to the number of voxels above a predefined threshold) because they rely on several other factors during LI assessment (for more details, see Refs. [33,36]).

## 4 LI and ROI selection

Obviously, LI values depend on the cortical volume used for their assessment. While some studies measure *Q*_LH_ and *Q*_RH_ across the whole hemisphere (i.e., global measures), others use ROIs (regional measures). Principally, language studies have focused on ROIs within the inferior frontal gyrus (Broca's area), prefrontal cortex, temporoparietal cortex, middle/superior temporal gyrus, angular gyrus, or fusiform gyrus (e.g., Refs. [38,42,45–47]). Although other studies have suggested that both global and regional ROIs yield concordant LI values (e.g., [38]), LI values with regional ROIs (frontal and temporoparietal) were found to correspond better with Wada scores than LIs with whole hemispheres [47]. Specifically, higher reliability for lateralization was obtained using ROI within the frontal lobe as compared to other temporoparietal regions (e.g., Refs. [46,48]; but see Ref. [42]).

**Fig. 3:**
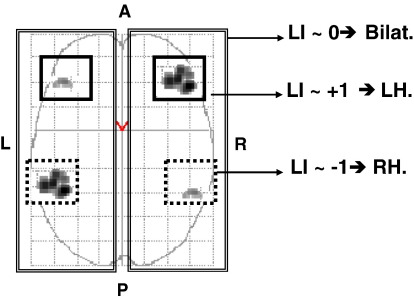
Influence of the ROI selection. Global ROI (whole hemisphere), frontal ROI and temporal ROI are illustrated on a schematic activation map.

Nevertheless, when computing one LI value per subject, the choice of ROI localisation and volume can lead to different conclusions about language laterality (see illustration in Fig. 3). This is particularly problematic in subjects with crossed language dominance. For example, Jansen et al. [49] reported a healthy normal subject performing a verbal fluency task, with LH dominance for frontal regions and RH dominance for temporal regions. Likewise, Baciu et al. [50] and Ries et al. [51] reported epileptic patients who had a negative LI value (right hemispheric dominance) in the frontal ROI and a positive LI value (left hemispheric dominance) in the temporal ROI.

Furthermore, the inclusion of the cerebellum is also delicate and is generally omitted when using whole hemisphere regions (but see Ref. [33]). This is due to the fact that some language components have a crossed cerebral and cerebellar representations of laterality (e.g., Ref. [52]), which may yield exaggerated bilateral laterality values. In addition, due to their localisation near the interhemispheric fissure, mesial regions are usually not considered when using global or regional ROIs for LI assessment, thereby limiting inferences concerning reorganisation mechanisms (i.e., intra- or interhemispheric) in brain-damaged patients when mesial regions are the best signatures of such mechanisms (e.g., supplementary motor area region, [53,54]).

Purposely, in order to depict a complete picture of language laterality for a given task, it might be more informative to use both regional (i.e., frontal and temporoparietal regions) and global ROIs for LI assessment in each subject.

## 5 LI threshold for hemispheric dominance

Hemispheric dominance is typically determined by the size of LI compared to a predefined threshold (LI_TH_) according to the following rule:

– LI>LI_TH_, left hemispheric dominance;
– LI<−LI_TH_, right hemispheric dominance;
– |LI|≤LI_TH_, bilateral dominance.

When *f* is held to 1, the threshold LI_TH_ value is usually set to 0.2 [46,55], but values like 0.1 [56], 0.15 [57], 0.25 [10,57] and even 0.3 [31] have also been used in previous work.

**Fig. 4:**
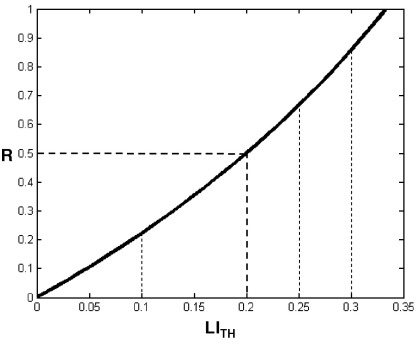
The relative difference (*R*) as function of the threshold (LI_TH_) on LI values (with *f*=1).

Consequently, language dominance is critically dependent on the LI_TH_ value, particularly when LI is compared to other clinical tests such as the Wada (e.g., [48,58]). As shown above in Eq. (3), LI_TH_ can be related to the relative ratio *R* between left and right contributions (*Q*_LH_ and *Q*_RH_, respectively). In this case, LI_TH_ could be chosen according to the desired *R* value. For instance, for left hemispheric dominance (i.e., LI>LI_TH_), *Q*_LH_ will be at least 50% more than *Q*_RH_ if LI_TH_ is set to 0.2. Fig. 4 illustrates the different possible values of LI_TH_ relative to *R* values. When using a fixed LI_TH_, the value 0.2 seems to be reasonable for attributing language dominance.

Instead of using an absolute and fixed LI_TH_ value, other groups have proposed variable and adapted LI_TH_ values that depend on the task and the group of subjects (e.g., Refs. [41,59]). Practically, all individual LI values are first assessed, and the mean (mean_LI_) and standard deviation (SD_LI_) are calculated. Then, the LI_TH_ value is set to mean_LI_−2SD_LI_ if mean_LI_>0, or to mean_LI_+2SD_LI_ if mean_LI_<0. This approach has been shown very useful when comparing patients to control subjects (e.g., is lateralisation in patients and controls identical?) by determining LI_TH_ according to the distribution of LI values in control subjects [39,41,60]. However, this approach is only appropriate for tasks with high lateralising power (i.e., high |LI| values) and showing low variability in controls (e.g., 2SD_LI_<|mean_LI_|).

## 6 LI and the statistical threshold

**Fig. 5:**
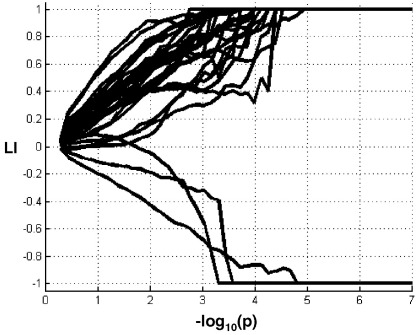
LI as a function of the threshold *P* (with *f*=1). Illustration with 30 subjects performing a semantic task.

One inherent issue in laterality assessment is the dependency between the statistical threshold and the LI value. This means that the LI value is not unique but can vary with the statistical threshold. Generally, the quantities *Q*_LH_ and *Q*_RH_ are determined at a specific threshold (e.g., number of activated voxels at *P*<.001, uncorrected, or *P*<.05, corrected). Different ways have been proposed to take into account the influence of statistical threshold on LI. A simple way is to compute LI at several thresholds [33,41,61]. However, several LI values per subject may not be easy to interpret. A more general approach is to assess the curves of LI(*P*) (i.e., LI as a function of the statistical threshold *P*) [23,46,59,62]. Fig. 5 illustrates LI(*P*) curves in 30 healthy subjects during a semantic decision task relative to perceptual matching, evidently with bilateral dominance (i.e., LI≅0) for low statistical thresholds and high LIs (in absolute value) for conservative thresholds. Using these curves, some authors have tried to find the best representative threshold to assess LI, for instance, by choosing a statistical threshold between the starting period (LI towards 0) and the plateau (LI towards ±1) (e.g., Refs. [23,59].

Moreover, Nagata et al. [62] have presented an alternative way to minimise the influence of the threshold on LI assessment. The idea is to identify empirically the dependency of the quantities *Q*_LH_ and *Q*_RH_ with the threshold (e.g., *z* score, *t* or *P* value) and then search for the best regression function to fit such dependency. For example, with individual *t* maps, we can write LI as:

(5) $$LI=f\cdot \frac{Q_{LH}(t)-Q_{RH}(t)}{Q_{LH}(t)+Q_{RH}(t)}=f\cdot \frac{A\cdot t^{\alpha }-B\cdot t^{\beta }}{A\cdot t^{\alpha }+B\cdot t^{\beta }}\text{.}$$

where *A*, *B*, *α* and *β* are constant values. By assuming that *α* and *β* are equal and fixed to a typical value of −4 [62], coefficients *A* and *B* are then determined by nonlinear regression, and thus, the LI value will be independent from the statistical threshold. Other values for *α* and *β* between −3 and −8 are also plausible depending on the data set and the contrast used for LI assessment. On the other hand, the quantities *Q*_LH_ and *Q*_RH_ may show different relationships with the threshold in some data sets (i.e., *α* and *β* are very different). In this case, other methods can alternatively be used (see below). Note that the regression function may depend on the statistical interval (e.g., range of *t* values) used during curve fitting, and it is critical to keep the same statistical interval during nonlinear regression when comparing LI between tasks or subjects.

In the same way, Branco et al. [39] have proposed a similar way to assess LI independently from the threshold using the estimated distribution of weighted *t* values in specific ROIs. This approach has been suggested to be less variable even in patients. Recently, a combined bootstrap/histogram analysis approach was proposed to generate threshold-free LI values [40]. By performing bootstrap analysis at different statistical thresholds within predefined ROIs, this study showed that LI assessment was more robust and stable compared to standard methods [40]. According to these reports, it is recommended to use one of these approaches that allow the influence of the statistical threshold on LI assessment to be minimised [39,40,62].

Alternatively, the threshold can be defined not on the statistical height but on the extent of activation [63]. In this case, for each subject, the threshold is adapted to obtain a certain predefined number of voxels and then LI is computed on these selected voxels (e.g., Refs. [36,64]). However, as the optimal number of voxels for a given task is usually unknown, the utility of this extent-based approach is limited.

## 7 LI and task selection

LI depends on the task chosen, even within a domain such as language (e.g., Refs. [36,59,61]). Thus, to obtain the best estimation of LI, previous studies have employed several tasks in the same subjects and then assessed LI for each task. In this way, each subject is characterised not only by one LI value but with a vector of LI values, which may be pertinent from a clinical perspective. On the other hand, clinicians may also be interested in estimating the best representative LI value over tasks for a given subject, particularly when confronting the LI value with other clinical or behavioural measures. In this case, an alternative approach is to analyse all tasks conjointly and combine these measures into one representative LI value. This combined task analysis approach, proposed by Ramsey et al. [61], was initially used with verb generation, synonyms generation, and categorisation tasks. Results indicated the strong lateralisation power of this approach and an improvement in the detection of language areas [61,65].

Several factors related to task selection may also influence language laterality. For instance, it has been suggested that task repetition can affect fMRI-based measures of language lateralization and lead to pseudoincreases in bilaterality [66]. Moreover, task difficulty can also affect the normal activity patterns with language paradigms (e.g., Refs. [67–70]), thereby perturbing language laterality particularly when LI is calculated across whole hemispheres. The input modality, visual or auditory, might also influence the hemispheric distribution of activated regions (e.g., Refs. [71,72], but see Ref. [73]). For instance, in healthy subjects and epileptic patients, LI values were found to be significantly stronger for visual than for auditory task presentation [74]. Other factors, including the use of words or pictures, silent or overt tasks, passive or active tasks and receptive and expressive language tasks, should also be taken into account when assessing LI within and between groups.

## 8 LI and baseline condition

Discrepancies in laterality assessment across studies may be attributable, in part, to differences in baseline tasks. The subtractive logic used in functional neuroimaging attributes the same “influence” to baseline and activation conditions. Different studies have emphasized the importance of baseline selection when interpreting functional maps (e.g., Refs. [75–77]). For instance, when a phoneme discrimination task was compared to three baseline conditions (rest, tone monitoring and passive listening), the baseline conditions systematically affected the amount of activation in different language areas [78], demonstrating different LI values with different baseline conditions. In addition, a recent study has investigated the most appropriate baseline task (picture naming, or passive viewing of nonsense objects) to isolate syntactic processes [79]. This showed that activity in language areas (e.g., Broca's area) was dependant on the baseline used, with more activation in these areas when the baseline was passive viewing of nonsense objects than picture naming. Moreover, using a low-level baseline condition (e.g., rest or fixation condition) may lead to LI values near to zero (i.e., more bilateral functional patterns), whereas a perceptual baseline may lead to higher LI values due to the elimination of nonrelevant regions involved in early perceptual and sensory processing [73]. Accordingly, control conditions that are close to the activation task might be more appropriate when nonrelevant low level areas need to be excluded from LI assessment [80]. Ideally, control conditions should have bilateral activation patterns (i.e., |LI|≅0) because if control activation has for example a strong RH dominance, then the activation task might be artificially lateralised to the LH (LI towards +1 as all right activations have been removed by the control condition).

## 9 Variability and reproducibility of LI

LI values are not absolute measures but can present some degree of variability across sessions and subjects. This is obviously related to the commonly observed variability of functional maps with language paradigms (e.g., Refs. [81,82]). Particularly, in the context of longitudinal fMRI studies, assessing the reliability and the reproducibility of LI values is imperative for the usefulness of such measures. For this aim, different studies have attempted to quantify the reproducibility of LI measures; however, as detailed above, these assessments are inherently related to the task used, the statistical threshold and the localisation of the regions of interest (e.g., Ref. [36]). For instance, recently, by computing laterality within inferior frontal and temporoparietal regions of interest for six different receptive and expressive language tasks, LI values were shown to be reproducible across sessions in both inferior frontal and temporoparietal regions [43]. In this study, the reproducibility of LI values was higher for (i) verb generation tasks, (ii) inferior frontal ROIs and (iii) *Q*_LH_ and *Q*_RH_ computed with *F* statistics rather than number of voxels [43]. In addition, for a wide range of statistical thresholds, LI values were compared across sessions, tasks, subjects and two a priori defined volumes of interest (classical language regions versus whole hemisphere) [65]. LI values were found to be reproducible for verb generation tasks and combined task analysis, with higher reproducibility when using regions of interest centred on language areas [65]. Moreover, LI reproducibility within and between session has been explored in a group of patients undergoing evaluation for epilepsy surgery [38]. LI had higher reliability within and across sessions when using one global (whole hemisphere) and three regional regions of interest (Broca's area, remaining prefrontal cortex, temporoparietal area).

## 10 Other considerations

Although it is possible to minimise the influence of these methodological factors with carefully conducted experiments, it is important to consider additional issues that might compromise the meaningfulness of the LI measure. Critically, the LI value is globally related to haemodynamic changes (i.e., physiological nature) detected by fMRI. Recently, Krach et al. [83] have shown that differences might exist between behavioural and physiological indicators of laterality, which might be problematic in some circumstances when confronting LI values with behavioural scores in patients. In addition, the risk of alteration of fMRI signal in the presence of brain insult (e.g., [84,85]) can lead to errors during the assessment of the quantities *Q*_LH_ and *Q*_RH_. Furthermore, the LI measure should be considered as representative of one facet of hemispheric dominance that is mainly related to gray matter activity. Other interesting facets are thus not taken into account in the LI measure, including, for instance, laterality of white matter pathways [86,87] and functional connectivity (e.g., Ref. [88]).

## 11 Conclusion

This review has highlighted the importance of controlling different methodological factors in order to ensure meaningful LI values. Without taking into account these factors, the LI measure might be seriously misguiding, which has, in some situations, discouraged some groups to use it and to rely on pure visual laterality rating (e.g., Refs. [14,89]). Critically, the quantification of *Q*_LH_ and *Q*_RH_, localisation of ROIs, task selection, the dependency on the statistical threshold and reproducibility are major issues that should be considered when using LI as a clinical measure for hemispheric dominance. I believe such factors warrant further investigations in order to establish a valid and unified methodological protocol for LI assessment with fMRI in different sensory and cognitive domains.
